# The relationship between mild alcohol consumption and mortality in Koreans: a systematic review and meta-analysis

**DOI:** 10.1186/s12889-015-2263-7

**Published:** 2015-09-18

**Authors:** Ji-Eun Park, Tae-young Choi, Yeonhee Ryu, Sung-Il Cho

**Affiliations:** Graduate School of Public Health, Seoul National University, Seoul, Republic of Korea; Korea Institute of Oriental Medicine, Daejeon, Republic of Korea; Institute of Health and Envirionment, Seoul National University, Seoul, Republic ofKorea

**Keywords:** Alcohol consumption, Alcohol, Drinking, Mortality, Systematic review, Meta-analysis, Korean

## Abstract

**Background:**

A recent systematic review reported that mild drinking showed beneficial effects on mortality. However, this relationship between alcohol consumption and mortality differs by race, and there are few studies on Koreans. In this study, we reviewed previous studies conducted on Koreans to investigate the association between mild drinking and mortality.

**Methods:**

Four databases (Medline, Web of Science, KoreaMed, and DBpia) were searched. Studies investigating the risk of alcohol consumption on three types of mortality (all-cause mortality, cancer-related mortality, and cardiovascular mortality) for Koreans were included.

**Results:**

A total of 16 studies assessed alcohol consumption as a risk factor for mortality. Nine studies reported on the risk of alcohol consumption in relation to all-cause mortality, eight to cancer-related mortality, and three to cardiovascular mortality. Among these, only studies assessing alcohol amount not drink status or drink frequency were included in meta-analysis. The results of the meta-analysis did not show a significant effect of mild alcohol consumption on all-cause mortality (5 studies, OR: 0.85, 95 % CI: 0.72, 1.01). While meta-analysis of studies using all-cancer mortality showed significant effect of alcohol consumption (4 studies, OR: 0.89, 95 % CI: 0.85, 0.94), results of studies including all-caner and specific type of cancer was not significant (7 studies, OR: 1.02, 95 % CI: 0.9, 1.15). Although a meta-analysis of cardiovascular mortality could not be conducted owing to a lack of studies, all studies reported a non-significant effect of occasional or mild alcohol consumption.

**Discussion:**

In this study, mild alcohol consumption in Korean did not show beneficial effect on mortality and it might be caused by three factors: criterion of mild drinking, the subjects, and sample size. The criterion of mild alcohol consumption was diverse in included studies. The effect of alcohol consumption could differ based on subjects’ sex, age as well as race. In addition, the effect of alcohol consumption might be different from previous one due to the small number of studies.

**Conclusions:**

Mild alcohol consumption did not show any beneficial effects in relation to all-cause, cancer-related, and cardiovascular mortality. Additional studies are necessary to verify any association between mild drinking and mortality in Koreans.

**Electronic supplementary material:**

The online version of this article (doi:10.1186/s12889-015-2263-7) contains supplementary material, which is available to authorized users.

## Background

Although alcohol abuse negatively affects health and mortality [1, 2], several studies reported that mild drinking has a beneficial effect on mortality, and depicted this relationship as a J-shaped curve [3–5]. In addition, recent meta-analyses have described how mild drinking had a beneficial effect on all-cause mortality [6], cardiovascular disease [7], and cancer-related mortality [8].

This association between alcohol and mortality might differ according to subjects’ characteristics. The dose of alcohol associated with protective effects on total mortality is lower among women than in men [6]. Rehm *et al.* reported a significant influence of drinking on mortality with a J-shaped association for males, but differences between drinking categories were much weaker for women [9]. In addition, the effect of alcohol might differ according to health status. A previous study reported that mild alcohol use may be beneficial for older adults in poor health, but not for those in good health [10]. Also, the effect of alcohol consumption on cardiovascular disease was different between men with and without hypertension [11].

Susceptibility to the effects of alcohol may also be contingent upon race [12]. Although mild alcohol consumption showed a beneficial effect on all-cause mortality in a previous study [6], in African Americans no J-shaped curve was found [13]. Meta-analysis of alcohol dose and total mortality reported a varying association between alcohol consumption and total mortality according to geographic region [6]. Although several studies have been conducted in East-Asian populations [14], there are few studies that focus specifically on Koreans. We therefore performed a systematic review to examine the relationship between mild alcohol consumption and mortality among Koreans.

## Methods

### Literature search and study inclusion criteria

We selected relevant published studies by searching Medline, Web of Science, KoreaMed, and DBPia databases up to September 30, 2014 without a restriction of study period. Search terms included “alcohol,” “mortality,” and “Korea.” All potentially eligible studies were considered for review, and the reference lists of included studies were examined. Only studies with Korean subjects were included. In addition, studies were eligible for inclusion only if they evaluated all-cause mortality, cancer-related mortality, or cardiovascular mortality as a result of alcohol consumption. When multiple articles had been published for a single study, the latest publication or study with more subjects was used. Two reviewers assessed relevant publications independently, and disagreements were resolved by a third reviewer. Extracted data included study design, study period, characteristics and number of participants, criteria for drinking, and the risk associated with alcohol consumption (PRISMA checklist - Additional file 1).

### Data synthesis

For this meta-analysis, studies in which the risk of alcohol consumption was based only on status (e.g., non-drinker/former drinker/current drinker) or frequency were excluded when analyzing the risk of mild drinking. To summarize the effects of alcohol on mortality, we extracted the risk estimates and 95 % confidence intervals (CI) from each study using the Cochrane Collaboration software, Review Manager (version 5.2. Copenhagen: The Nordic Cochrane Centre, The Cochrane Collaboration, 2012).

Although there is a consensus on moderate drinking as constituting up to one drink per day for women and up to two drinks per day for men [15], the range of alcohol intake showing a protective effect in previous meta-analyses was variable [6–8]. For this reason we compared the risk of non-drinkers and mild drinkers consuming the least alcohol, based on each study’s criteria. Chi-square, tau^2^, and Higgins I^2^ tests were used to assess heterogeneity. When notable heterogeneity was present (I^2^ index ≥ 80 %), a random-effects model was used.

### Quality assessment and publication bias

Two independent reviewers critically appraised the methodological quality of included studies using the Newcastle-Ottawa scale. The Newcastle-Ottawa scale is a quality assessment tool based on selection of cases and controls (0–4 points for case–control studies and 0–6 points for cohort studies), comparability (0–2 points), and exposure (0–4 points in case–control studies) or outcome (0–5 points in cohort studies). We defined the studies with less than 4 points in case–control studies and less than 6 points in cohort studies as low quality, and these were excluded from the meta-analysis.

## Results

**Fig. 1:**
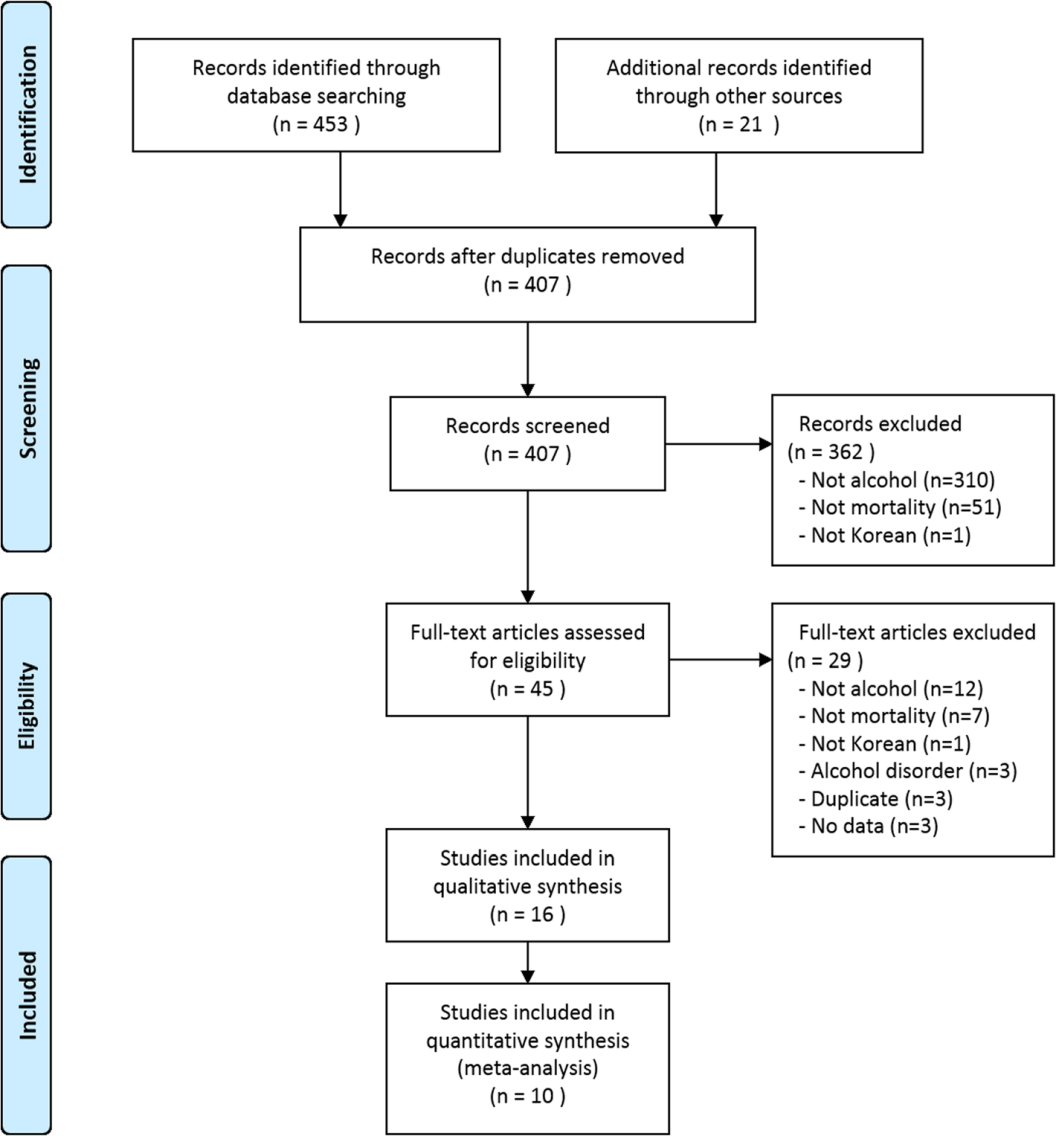
Flowchart of the literature search

Of a total of 474 identified studies, 429 were excluded after reviewing article titles. Based on a review of abstracts another 29 studies were excluded, and 16 fulfilled the inclusion criteria (Fig. 1). Of 29 studies, 12 studies did not related to alcohol, 7 did not assess mortality, and subjects did not meet inclusion criteria in one study. We excluded three studies investigating mortality associated with alcohol disorder [2, 16, 17], because it is a disease and is not appropriate in the assessment of the effects of typical alcohol use. In addition, we excluded three studies because they used the same participants as other studies [18–20], and another three studies that did not include appropriate data [21–23].

**Fig. 2:**
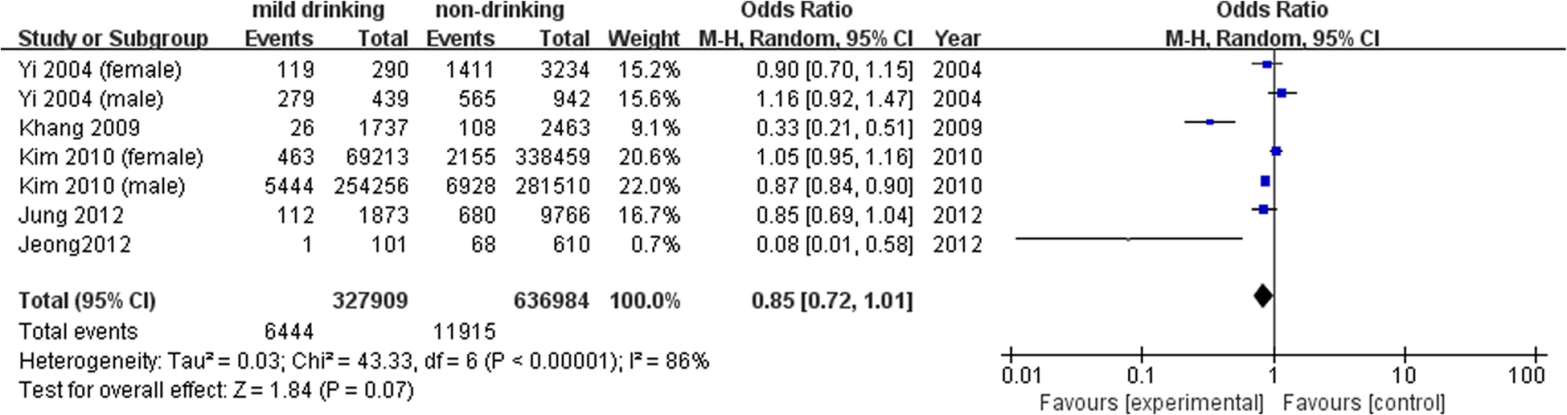
Pooled results of mild drinking on the risk of all-cause mortality

The characteristics of included studies are summarized in Table 1. Of the 16 studies, five reported on all-cause mortality, six on cancer-related mortality, and one on cardiovascular mortality. Two studies reported all-cause and cancer-related mortality, and the remaining two reported on all-cause and cardiovascular mortality. Ten of the 16 studies used weekly or daily amount as the measure of alcohol consumption, three used drinking frequency, and three used drinking status. A category of no alcohol intake was the reference category in 15 studies, and one study used a mild alcohol group as reference [24]. Duration of follow-up ranged from 1 to 20.8 years, and sample size varied from 910 to 1,341,393 in the 16 studies.

**Table 1:** Characteristics of studies on alcohol drinking and mortality

First author (Year)	Study design	Study period (Follow-up duration)	Participants	Number of cases (death)	Measures of alcohol consumption	Risk estimate (Confidence Interval)		Criteria of mild drinking
All cause mortality								
Kim 2007 [25]	Cohort	1995-2001 (6 years)	Aged ≥20	3,366 (228)	Status	Men	Women	n.r
						None: 1.0	None: 1.0	
						Former:1.01 (0.57-1.77)	Former: 1.41 (0.62-3.05)	
						**Current:0.75 (0.47-1.22)**	**Current: 1.69 (1.01-2.98)**	
Rhee 2012 [26]	Cohort	1993-2008 (15 years)	Men aged 40-59	14,533 (990)	Status	None; 1.0		n.r
						Former: 1.17 (0.98-1.39)		
						**Current: 1.40 (1.07-1.83)**		
Park 1999 [27]	Nest case–control	1993-1997 (4–5 years)	Aged ≥40	38,496 (19,258)	Frequency	None: 1.0		n.r
						**Occasional: 1.0 (0.94-1.06)**		
						Frequently: 1.17 (1.1-1.26)		
Sull 2009 [1]	Cohort	1985-2005 (20.8 years)	Men aged ≥55	2,624 (1,984)	Frequency	None: 1.0		n.r
						**Few time a month** ^**a**^ **: 1.03 (0.89-1.2)**		
						Few time a week^**b**^: 1.04 (0.93-1.16)		
						Daily: 1.25 (1.10-1.43)		
Yi 2004 [28]	Cohort	1985-1999 (14 years)	Aged ≥55	6,292 (2,673)	Weekly amount	Men	Women	≤70 g/week
						None; 1.0	None: 1.0	
						**Low: 1.06 (0.92-1.23)**	**Low: 0.94 (0.77-1.15)**	
						Moderate:1.09 (0.96-1.23)	Moderate:1.16 (0.77-1.74)	
						Heavy: 1.35 (1.14-1.60)		
Khang 2009 [30]	Cohort	1998-2001 (3 years)	Aged ≥30	8,366 (310)	Monthly amount	None: 1.0		n.r
						Former: 2.03(1.42-2.91)		
						**Minimal: 0.60 (0.39-0.93)**		
						Q1: 1.04 (0.62-1.68)		
						Q2: 1.09 (0.71-1.67)		
						Q3: 1.17 (0.78-1.76)		
						Q4: 1.23 (0.82-1.84)		
Kim 2010 [31]	Cohort	2000-2005 (5 years)	Aged 40-69	1,341,393 (19,375)	Daily amount	Men	Women	<30 g/day for men
						None: 1.0	None: 1.0	<15 g/day for women
						**1-14.9 g: 0.87 (0.84-0.91)**	**1-14.9 g: 0.99 (0.85-1.15)**	
						15-29.9 g:0.88 (0.84-0.92)	≥15 g: 1.39 (1.08-1.79)	
						30-89.9 g:1.07 (1.02-1.13)		
						≥90 g: 1.29 (1.22-1.37)		
Jeong 2012 [29]	Cohort	2005-2006 (1 year)	Aged ≥65	997 (113)	Weekly amount	None: 1.0		≤7drinks/week
						**Light: 0.08 (0.01-0.58)**		
						Moderate: 1.15 (0.46-2.85)		
						Heavy: 1.44 (0.81-2.56)		
Jung 2012 [24]	Cohort	1993-2004 (11 years)	Aged ≥20	16,320 (1,122)	Weekly amount	None: 1.18 (0.96-1.45)		≤90 g/week
						**0.1-90 g: 1.0**		
						90.1-252 g: 1.29 (0.99-1.66)		
						252.1-504 g: 1.31 (1.00-1.71)		
						≥504.1 g: 1.39 (1.05-1.83)		
Cancer-related mortality								
Lim 2008 ^c^ [32]	Cohort	1993-1996 1995–2002 (2–7 years)	Aged ≥65	14,304 (112)	Status	None:1.0		≤24 g/day
						Ex: 1.47 (0.87-2.47)		
						**Current: 0.83 (0.53-1.31)**		
Lee 2002 [34]	Cohort	1985-1998 (13 years)	Aged ≥55	2,681 (253)	Weekly amount	None: 1.0		1-4 times/month
						**Light: 0.98 (0.64-1.50)**		
						Moderate: 1.01 (0.69-1.48)		
						Heavy: 1.2 (0.89-1.62)		
Jee 2004 ^d^ [36]	Cohort	1993-2002 (7–9 years)	Aged 30-95	1,283,112 (3,807)	Daily amount	Men	Women	1-24.9 g/day
						None: 1.0	None: 1.0	
						**1-24.9 g: 1.0 (0.9-1.1)**	**Drinker: 1.2 (0.9-1.5)**	
						25-49.9 g: 1.0 (0.9-1.2)		
						50-99.9 g: 1.1 (0.9-1.4)		
						≥100 g: 1.4 (1.0-1.8)		
Park 2006 [35]	Cohort	1996-2004 (9 years)	Men aged ≥ 20	14,578 (7,271)	Weekly amount	None: 1.0		n.r
						**1-124.1 g: 0.94 (0.88-1.00)**		
						≥124.2 g: 1.05 (0.98-1.12)		
Kim 2010 [31]	Cohort	2000-2005 (5 years)	Aged 40-69	1,341,393 (19,375)	Daily amount	Men	Women	<30 g/day for men
						None: 1.0	None: 1.0	<15 g/day for women
						**1-14.9 g: 0.91 (0.85-0.97)**	**1-14.9 g: 0.99 (0.85-1.15)**	
						15-29.9 g: 0.93 (0.87-1.0)	≥15 g: 1.39 (1.08-1.79)	
						30-89.9 g:1.06 (0.98-1.15)		
						≥90 g: 1.21 (1.11-1.33)		
Kimm 2010^e^ [33]	Cohort	1993-2006 (14 years)	Aged 30-93	782,632 (996)	Daily amount	None: 1.0		≤24 g/day
						**1-24 g: 1.9 (1.6-2.3)**		
						25-49 g: 2.7 (2.1-3.5)		
						50-99 g: 3.7 (2.8-5.0)		
						≥100 g: 3.4 (2.2-5.3)		
Yi 2010 ^f^ [37]	Cohort	1985-2005 (20.8 years)	Aged ≥55	6,291 (360)	Weekly amount	Men	Women	<138 g/week for men, <12 g/week for women
						None: 1.0	None: 1.0	
						**Low: 1.18 (0.83-1.69)**	**Low: 1.15 (0.53-2.51)**	
						Moderate:1.06(0.73-1.56)	High: 1.63 (0.83-3.19)	
						High: 1.26 (0.88-1.82)		
Jung 2012 [24]	Cohort	1993-2004 (11 years)	Aged ≥20s	16,320 (1,122)	Weekly amount	None: 1.55 (1.15-2.11)		≤90 g/week
						**0.1-90 g: 1.0**		
						90.1-252 g: 1.7 (1.16-2.49)		
						252.1-504 g: 1.84 (1.24-2.72)		
						≥504.1 g: 2.07 (1.39-3.09)		
Cardiovascular mortality								
Meng 1987 [38]	Case–control study	1982-1983	Aged 35-65	910 (190)	Frequency	None: 1.0		1-2 times/month
						**1-2 times/month: 0.92**		
						1-2 times/week: 1.09		
						3-4 times/week: 2.27		
						Everyday: 2.17		
Sull 2009 [1]	Cohort	1985-2005 (20.8 years)	Men aged ≥55	2,624 (1,984)	Frequency	None: 1.0		n.r
						**Few times a month** ^**a**^ **: 0.98 (0.67-1.42)**		
						Few times a week^**b**^: 1.06 (0.82-1.37)		
						Daily: 1.36 (1.0-1.84)		
Yi 2004 [28]	Cohort	1985-1999 (14 years)	Aged ≥55	6,292 (672)	Weekly amount	Men	Women	≤70 g/week
						None: 1.0	None: 1.0	
						**<70 g: 0.98 (0.69-1.37)**	**<70 g: 0.92 (0.61-1.38)**	
						70-503.9 g: 1.06 (0.8-1.39)	≥70 g: 0.89 (0.33-2.4)	
						≥504 g: 1.52 (1.06-2.19)		

BP: blood pressure, FBS: fasting blood sugar, n.r: not reported, ^a^: almost daily plus 2 to 3 times a week, ^b^: 1 to 4 times a month, ^c^: Colorectal cancer, ^d^: Hepatocellular carcinoma, ^e^: Esophageal cancer, ^f^: Digestive cancer. The group in bold font was analyzed in review or meta-analysis

### Mortality

1. All-cause mortality
  Of the two studies using drinking status as a criterion, one reported a significantly high risk only among women [25], and the other showed a significant effect on mortality in current drinkers compared with non-drinkers [26]. Two studies using frequency as a drinker classification criterion showed no significant results [1, 27]. In the five studies using amount of alcohol consumed, mild drinkers showed no significant mortality risk in two studies [24, 28], while three reported a significantly lower risk among men [29–31].
  To analyze the risk of mild drinking, only five studies measuring the amount of alcohol consumed were included in the meta-analysis. The results of the meta-analysis did not show favorable effects of mild alcohol drinking on total mortality (OR: 0.85, 95 % CI: 0.72, 1.01) (Fig. 2).
2. Cancer-related mortality
  Of eight studies in total, one study assessed drinking status and seven studies assessed alcohol amount. The study using drinking status showed non-significant results [32]. Of seven studies using alcohol amount for mild drinking classification, three reported significant results. Although Kimm *et al.* reported high mortality in mild drinkers [33], another two studies found lower mortality in mild drinkers compared with non-drinkers [24, 31].
  Four reported the effects of alcohol consumption on all mortality from cancer [24, 31, 34, 35], and another four assessed the effect of alcohol on hepatocellular carcinoma [36], colorectal cancer [32], esophageal cancer [33], and digestive cancer [37]. Pooled results of mild drinking from four studies using all-cancer mortality showed beneficial effect (OR: 0.89, 95 % CI: 0.85, 0.94), however, it was not significant when adding three studies [33, 36, 37] assessing risk of mild drinking on specific type of cancer (OR: 1.02, 95 % CI: 0.90, 1.15) (Fig. 3).

**Fig. 3:**
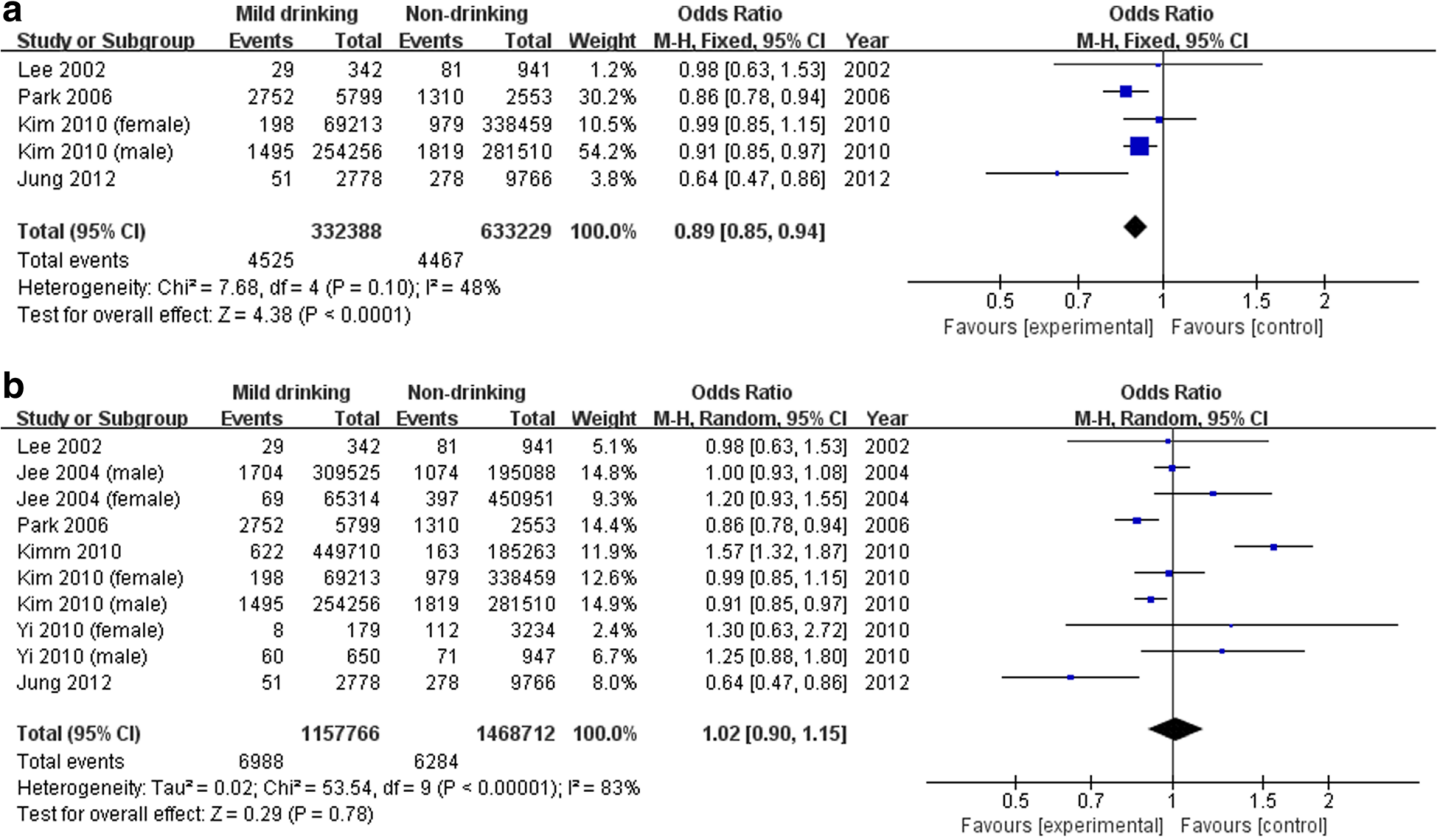
Pooled results of mild drinking on the risk of cancer-related mortality. **a** All-cancer mortality. **b** Mortality from all-cancer and specific type of cancer
3. Cardiovascular mortality
  Three studies assessed the cardiovascular risk related to drinking alcohol. Although two studies using frequency and one study using alcohol amount as drinking criterion reported lower cardiovascular mortality in occasional or mild drinkers compared with non-drinkers [1, 28, 38], none of the results were statistically significant. Owing to the lack of studies, a meta-analysis of mild drinking as a risk factor for cardiovascular mortality could not be conducted.

### Quality assessment and publication bias

Overall, the methodological quality of the included studies was moderate to high. Scores on the Newcastle-Ottawa scales were 4 to 5 points in case–control studies and 6 to 9 points in cohort studies. Exposed and non-exposed groups were in the same community in all studies, and most studies used structured interviews to ascertain exposure data. Additionally, all cohort studies used independent blind assessment or record linkages to assess outcomes. Based on the results of the quality assessment, none of studies was excluded from the meta-analysis.

**Fig. 4:**
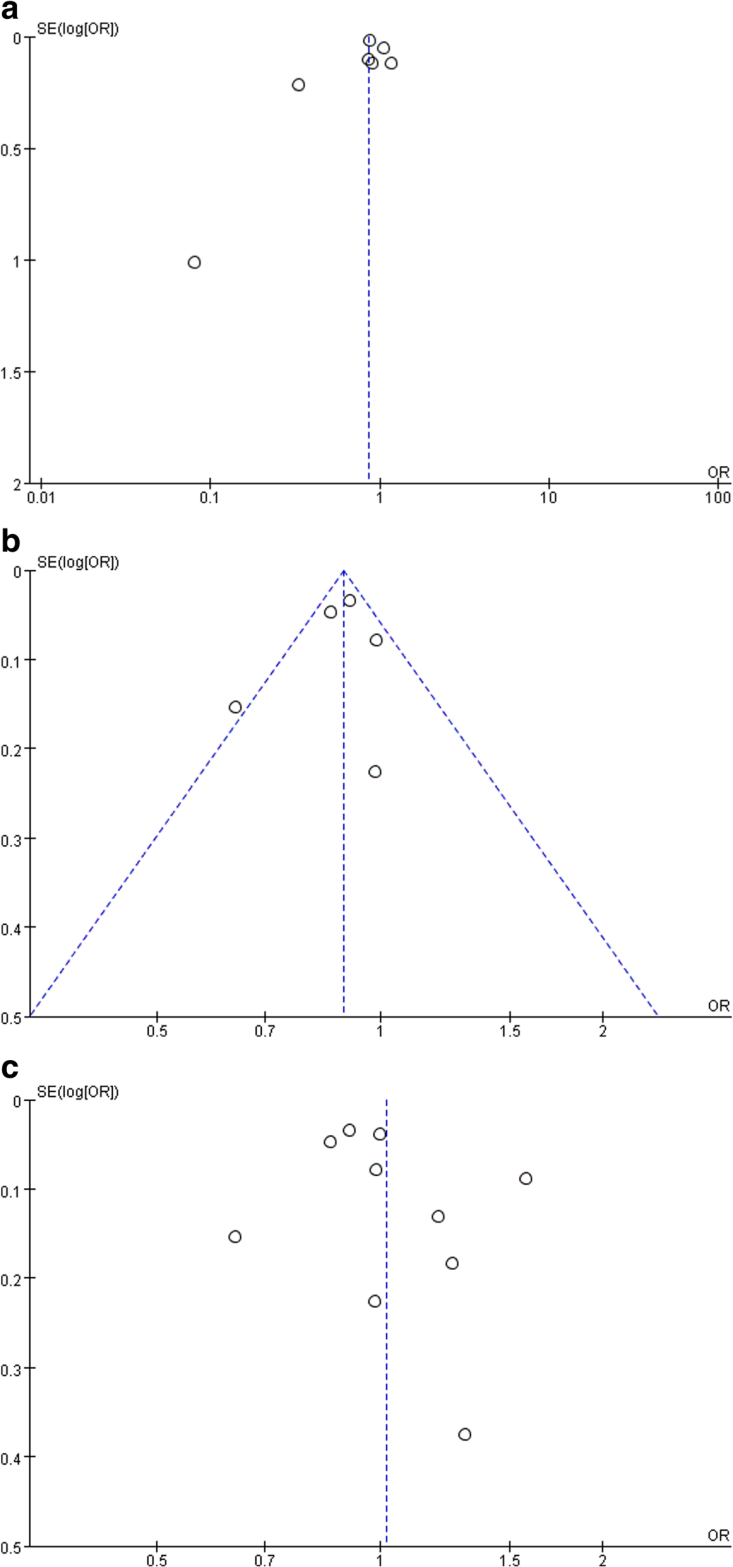
Funnel plot for alcohol consumption in relation to all-cause mortality and cancer-related mortality. **a** All-cause mortalit`y. **b** All-cancer mortality. **c** Mortality from all-cancer and specific type of cancer

The funnel plot did not present potential for publication bias (Fig. 4). Owing to the small number of studies for each outcome, a statistical test to evaluate publication bias could not be conducted.

## Discussion

In recent meta-analyses, mild alcohol consumption showed a beneficial effect on all-cause mortality [6], cardiovascular mortality [7], and cancer-related mortality [8]. However, in this study mild drinking did not demonstrate a protective effect for all-cause mortality and cardiovascular mortality. Although mild alcohol consumption showed significant effect on all-cancer mortality, it was not significant on cancer-related mortality when adding various type of cancer. The difference in results could be caused by three factors: the criteria used to define mild drinking, the subjects, and the sample size.

First, the criterion of mild drinking is diverse. The previous meta-analyses of the relation between alcohol and cardiovascular and cancer-related mortality reported a beneficial effect from 2.5 to 14.9 g/day [7] and less than 12.5 g/day [8], respectively. The criterion used to define a mild amount of alcohol is inconsistent among studies included in this review. In the study by Jung *et al.*, a weekly amount of less than 90 g was considered as mild drinking [24], while less than 70 g was used in the Yi *et al.* study [28]. Moreover, Kim *et al.* defined mild drinking as a daily amount of 30 g [31], whereas Jee *et al.* designated 25 g/day [36] and Lim and Park preferred 24 g/day [32]. Unjustified categorization of alcohol consumption might cause inaccurate results in individual studies, and different criteria for mild alcohol consumption between studies make it difficult to compare the results. To accurately assess the effect of alcohol consumption, a consensus on the alcohol intake considered to represent “mild drinking” should first be reached.

Second, the subjects included in this meta-analysis and those in previous ones differed. This review included only the Korean population. Besides biological factors including race, the effect of alcohol drinking could also differ based on behavioral factors [39]. While total alcohol per capita in Korea was higher, at 12.3 L of pure alcohol (men: 21, women: 3.9), compared with the world average of 6.2 L, the proportion of heavy episodic drinking was lower (6.0 %) than the world average (7.5 %) [40]. Not only the amount of alcohol and risky drinking, but also the type of beverage has an influence on the effect of alcohol on mortality. Although previous studies reported that wine and beer showed a greater protective effect than spirits on cardiovascular disease and cancer [41, 42], beer and wine accounted for only 26.6 % of total alcohol consumption in Korea [40]. Further studies assessing the effect of alcohol consumption should consider factors such as drinking patterns and beverage type.

Lastly, there is a possibility that the different results were due to the small number of studies. Whereas previous reviews included between 18 and 84 studies, the number of studies in this review was less than 10 for each outcome. Moreover, the studies including fewer than 10,000 subjects numbered 8 of the total 16 studies. To understand the reasons for these different results, more studies including Korean participants should be conducted to investigate the association between mild alcohol consumption and mortality risk.

Most of the included studies used non-drinkers as a reference group, but it is unclear whether they graded former drinkers as non-drinkers. Because some former drinkers quit drinking for health reasons, analyzing these subjects as non-drinkers could lead to biased results. Further misclassification, for example including occasional drinkers as non-drinkers or low-level drinkers, could bias risk estimates [43]. Appropriate classification of drinkers is important in assessing the risks of alcohol consumption.

In this review, several studies used different criteria for men and women [31, 37], while others applied the same criteria and analyzed both sexes together. In a previous meta-analysis investigating alcohol and total mortality, 2 to 4 drinks per day for men and 1 to 2 drinks per day for women were inversely associated with total mortality [6]. Women may be more vulnerable to alcohol-related risk, and men and women exhibit different drinking patterns [40]. Participants’ characteristics, such as sex, should be considered when assessing the impact of alcohol.

The age of the subjects in each study was diverse. Moreover, several studies chose subjects according to their residential area [28, 37] while others enrolled participants based on health examination [31]. Such variations might have contributed to population heterogeneity in this meta-analysis.

Previous studies have attributed the apparent benefits of alcohol to antioxidant capacity, anti-inflammatory effects, and the change in lipid profiles [41]. Rimm *et al.* reported that alcohol intake is causally related to a lower risk of coronary heart disease through changes in lipids and hemostatic factors [44]. Furthermore, another study revealed that alcohol has anti-inflammatory effects by reducing plasma fibrinogen and interleukin-1α levels [45]. However, high-dose ethanol increases mortality [6], and Carnevale and Nocella reported that long-term alcohol consumption involves increased oxidative stress and the production of pro-inflammatory cytokines and adhesion molecules [46]. The biological mechanism of alcohol on health and mortality should be further assessed through additional studies.

## Conclusions

This study did not provide evidence for the beneficial effects of mild drinking on all-cause, cancer-related, and cardiovascular mortality. Given the small number of studies included, larger prospective studies of the Korean population with more consistent criteria regarding mild drinking are needed.

## Additional file

Additional file 1:
**PRISMA guideline.** (DOC 64 kb)
